# Interpretable prediction on treatment response of piperacillin–tazobactam for lower respiratory tract infections using machine learning

**DOI:** 10.1097/MD.0000000000043460

**Published:** 2025-08-01

**Authors:** Zhijing Zhu, Tao Yang, Kun Han, Xinjuan Liu, Yemeng Yang, Likun Pan

**Affiliations:** a School of Materials and Chemistry, University of Shanghai for Science and Technology, Shanghai, China; b Department of Pharmacy, Shanghai Pudong Hospital, Fudan University Pudong Medical Center, Shanghai, China; c Shanghai Key Laboratory of Magnetic Resonance, School of Physics and Electronic Science, East China Normal University, Shanghai, China; d Department of Respiratory and Critical Care Medicine, Shanghai Pudong Hospital, Fudan University Pudong Medical Center, Shanghai, China.

**Keywords:** antimicrobial use, lower respiratory tract infections, machine learning, piperacillin–tazobactam

## Abstract

Empiric piperacillin/tazobactam (PIPT) therapy is commonly used in high-risk patients with severe community-acquired pneumonia. However, its overuse may lead to antibiotic resistance and unwanted side effects. To this end, we developed and validated a machine learning (ML) model to assess the effectiveness of PIPT in the treatment of septic lower respiratory tract infections and to explore relevant influencing factors. The study was based on data from hospitalized patients treated with PIPT, and a dataset of bacterial lower respiratory tract infections was constructed by retrospective analysis and divided into training and testing sets in a 7:3 ratio. After screening the key predictors using least absolute shrinkage and Least Absolute Shrink age and Selection Operator regression methods, 5 ML models, including logistic regression and random forest, were used to train these factors to predict efficacy. Model interpretation was performed using the SHapley Additive exPlanations technique. The results showed that the decision tree model had a performance score of 0.73 (95% CI 0.61–0.86) for prediction. The SHapley Additive exPlanations analysis identified several important factors for treatment failure, including low serum albumin levels, reduced drug dosage, and comorbidities such as chronic obstructive pulmonary disease and heart failure, in addition to an unfavorable neutrophil-to-lymphocyte ratio of ≥70%. This study demonstrates that the ML model is effective in predicting the outcome of PIPT therapy and helps to personalize medical regimens while adjusting strategies by identifying high-risk individuals, ultimately achieving the dual goals of optimizing patient care and reducing inappropriate antibiotic use.

## 1. Introduction

Lower respiratory tract infections (LRTIs), which include pneumonia, bronchiectasis, bronchitis, and tracheitis, are common infectious diseases. The main types of LRTIs are community-acquired pneumonia, hospital-acquired pneumonia, pneumonia in immunocompromised hosts, acute exacerbations of chronic obstructive pulmonary disease (COPD), and bronchiectasis with infection. These infections present with diverse clinical manifestations and are caused by a wide variety of microorganisms,^[1]^ LRTIs are among the leading causes of infectious disease-related deaths globally. According to World Health Organization estimates, in 2019, LRTIs ranked fourth in both global mortality and disability-adjusted life years, making them a critical public health issue that needs urgent attention.^[2]^

Amoxicillin and tetracycline are widely used in the clinical treatment of LRTIs due to their low incidence of adverse reactions, making them the preferred antibiotics for many cases. However, in patients with severe community-acquired pneumonia who are at risk for Pseudomonas aeruginosa or other resistant Gram-negative bacteria,^[3]^ the 2019 guidelines from the Infectious Diseases Society of America and the American Thoracic Society recommend that initial empirical therapy include anti-pseudomonal β-lactam antibiotics, such as piperacillin/tazobactam (PIPT). This recommendation is also applicable to patients with moderate to severe acute exacerbations of COPD or bronchiectasis, who are at risk of Pseudomonas aeruginosa infection.

In the clinical management of LRTIs, current laboratory testing methods often fail to provide timely and reliable pathogen identification. As a result, empirical antibiotic therapy plays a critical role in the early stages of treatment. However, inappropriate use of empirical PIPT therapy is common in cases of severe bacterial infections.^[4]^ It is estimated that up to 50% of antimicrobial prescriptions are unnecessary,^[5]^ contributing to the overuse of broad-spectrum antibiotics. This overuse not only disrupts the beneficial microbiota, causing side effects, but also fosters the development of drug-resistant bacteria. The rise of resistant pathogens complicates infection treatment, increases healthcare costs, and may lead to higher patient mortality, ultimately undermining infection prevention and control efforts.

Antibiotic resistance evolves through interconnected mechanisms, with patient-specific factors critically influencing outcomes. These include physiological alterations such as decreased serum albumin (ALB) levels and pathophysiological changes from comorbidities, including COPD and heart failure (HF), that modify tissue drug penetration.^[6,7]^ Clinical practice factors, such as inappropriate prescribing and subtherapeutic dosing, create selective pressure for resistant organisms.^[8]^ Host immunocompetence, quantifiable through inflammatory markers like the neutrophil-to-lymphocyte ratio (NLR), significantly affects antimicrobial efficacy.^[9]^ Therefore, accurately predicting the necessity and potential efficacy of broad-spectrum antibiotics like PIPT before treatment initiation is crucial for preventing unnecessary antimicrobial use and mitigating the spread of resistant bacteria.^[10]^

Machine learning (ML) is an effective tool for analyzing large amounts of data to find the relationships between input and the desired output, and has been used widely in different fields of science.^[11–14]^ Moran et al^[15]^ used the eXtreme Gradient Boosting (XGBoost) model to predict the resistance of gram-negative bacteria in the blood and urine of hospitalized patients to compound amoxicillin and PIPT. This approach aimed to guide the selection of antimicrobial drugs and reduce the inappropriate use of broad-spectrum antibiotics in hospitals. However, their work did not give an interpretable analysis of the ML model, which diminished the model’s utility in personalized treatment.

The efficacy of PIPT treatment for LRTIs is known to be associated with patients’ gender, age, physical condition, infection, and medication information.^[16–18]^ Therefore, in this work, ML was employed to predict the treatment efficacy of empirically prescribed PIPT based on patients’ clinical information. Importantly, this work emphasizes the interpretability of the ML model, enhancing its practical application in personalized therapy, and thus exhibits high promise for helping clinicians to make informed decisions about antibiotic treatment decisions for LRTI patients and facilitate personalized therapy.

## 2. Methods

### 2.1. Study population

Adult patients diagnosed with LRTIs or related infection (confirmed through a comprehensive assessment of patient symptoms, signs, laboratory parameters, and pulmonary imaging), followed by discharge from the hospital’s respiratory ward; initial treatment with PIPT (2.5 g, 3 times daily) or (3.125 g, 3 times daily) as the preferred medication regimen. This dosage regimen reflects the standardized medication protocol established at Shanghai Pudong Hospital for the management of severe bacterial LRTIs in critically ill patients. The hospital’s dosing strategy takes into account local patient characteristics, such as (e.g., body weight, renal function, drug tolerance), as well as clinical outcomes observed in prior cases. This protocol aims to balance therapeutic efficacy with a reduced risk of adverse effects. The exclusion criteria were as follows: incomplete clinical data; inability to assess treatment efficacy (such as death within 3 days of treatment initiation, transfer to another facility, or development of acute respiratory distress syndrome); concurrent infections in other anatomical sites; changes in medication type or dosage during treatment; cases with identified pathogens or diagnosed viral or atypical pathogen infections.

### 2.2. Therapeutic efficacy of piperacillin–tazobactam

The primary outcome was the therapeutic efficacy of empirically prescribed PIPT upon admission, categorized as effective or ineffective. Efficacy was evaluated after at least 3 days of treatment based on the following criteria: Clinical signs and symptoms, reduction of fever (body temperature ≥ 37.3°C), improvement in abnormal lung sounds on auscultation (wheezing or crackles), and improvement in mental status (enhanced alertness or reduced confusion); Laboratory parameters, decrease in elevated white blood cell count towards normal range and significant reduction in C-reactive protein levels; Radiological findings, improvement in chest radiography or computed tomography scans indicated by resolution or reduction of lung infiltrates. Patients demonstrating significant improvement in any of these indicators were classified as having effective treatment, justifying discontinuation or reduction of antibiotic use. Those without significant improvement were considered to have ineffective treatment, necessitating escalation of antibiotic therapy or patient transfer.

### 2.3. Data collection

In this study, feature selection focused primarily on patient characteristics and treatment factors, including demographic parameters, clinical parameters, and the dosage of PIPT. These features were chosen because the study aimed to evaluate the initial efficacy of empirical therapy in critically ill patients, where microbiological data are often unavailable at the onset of treatment. Although microbiological factors, such as pathogen identification and antibiotic susceptibility, significantly impact treatment outcomes, they were not included in the analysis because the study focused on assessing the effectiveness of empirical therapy before microbiological results become available. During the feature selection process, potential confounding factors that might influence treatment outcomes were considered, including patients’ clinical status, comorbidities, and medication dosages. For potential confounders not included in the analysis, such as microbiological data, strict inclusion and exclusion criteria were applied to ensure relative homogeneity of the study population. Additionally, the multivariate-adjusted least absolute shrinkage and selection operator (LASSO)^[19]^ regression model was employed to adjust for patient characteristics and treatment factors, thereby reducing the impact of confounding bias.

### 2.4. Variables

In this study, a total of 22 factors were used as candidate covariates to predict PIPT treatment outcomes in LRTIs. These covariates covered a variety of aspects, including patient demographics, infection-related parameters, as well as details of antimicrobial therapy and combined antimicrobial therapy (Table 1).

**Table 1:** Clinical variables used in the study.

Characteristics	Abbreviations	Assignments
Gender	Gender	Female = 0; Male = 1
Age	Age	Continuous variable
Weight	Weight	Continuous variable
Estimated glomerular filtration rate	EGFR	Continuous variable
Blood urea nitrogen	BUN	<11 mmol/L = 0; ≥11 mmol/L = 1
Serum albumin	ALB	≥35 g/L = 0; <35 g/L = 1
Body temperature	Temp	≥37.3°C = 1; <37.3°C = 0
White blood cell count	WBC	≥9.5 × 10^−9^ = 1; <9.5 × 10^−9^ = 0
C-reactive protein	CRP	≥10.0 mg/L = 1; <10.0 mg/L = 0
Neutrophil-to-lymphocyte ratio	NLR	≥75% = 1; <75% = 0
Respiratory failure	RF	Yes = 1; no = 0
Purulent sputum	PS	Yes = 1; no = 0
Recent hospitalization history (in 1 mo)	RH	Yes = 1; no = 0
Active tumor	CA	Yes = 1; no = 0
Chronic obstructive pulmonary disease	COPD	Yes = 1; no = 0
Bronchiectasis	BE	Yes = 1; no = 0
Interstitial lung disease	ILD	Yes = 1; no = 0
Cerebrovascular disease	CVD	Yes = 1; no = 0
Heart failure	HF	Yes = 1; no = 0
Outpatient treatment history before admission	OPTH	Yes = 1; no = 0
Dosage of piperacillin/tazobactam	PIPD	2.5*g*, *Q*8*H* = 1; 3.125*g*, *Q*8*H* = 0
Combination antimicrobial therapy	CAT	Yes = 1; no = 0

### 2.5. Statistical analysis

The clinical data of patients were presented as continuous or categorical variables. The Shapiro–Wilk test was utilized to assess distribution normality. Data were reported as mean ± SD and median (interquartile range) for variables of normal and non-normal distributions, respectively. For distribution comparison, *t* test and Wilcoxon rank-sum test were used as appropriate. Categorical data were expressed as numbers and frequencies and compared with the Chi-square test. A *P* value < 0.05 indicated statistical significance. Python (version 3.9.18, Python Software Foundation, Beaverton ) and Scikit-learn (version 1.3.0, https://scikit-learn.org) were used for all analyses in this study.

### 2.6. ML analysis

**Figure 1.**
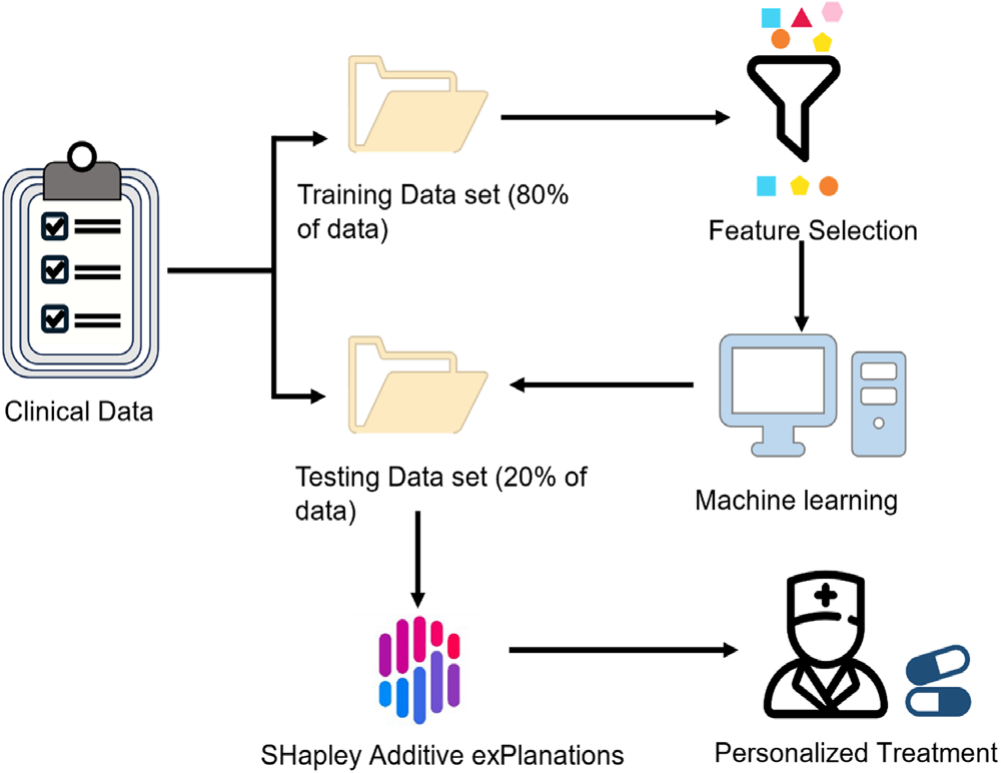
Clinical workflow of using ML algorithms to predict the treatment efficacy of PIPT for LRTIs. LRTIs = lower respiratory tract infections, ML = machine learning, PIPT = piperacillin/tazobactam.

First, we utilized stratified random sampling to partition the dataset into a training set and an independent test set with a ratio of 4:1. Following this, continuous variables, including age, weight, and estimated glomerular filtration rate, were normalized with the min-max normalization. Then, LASSO regression was used to identify features relevant to treatment response. Five ML models, including logistic regression (LR), random forest (RF), decision tree (DT), Gradient Boosting Decision Tree, and XGBoost, were constructed to predict the therapeutic efficacy of PIPT treatment for LRTI patients. The performance of the models was evaluated with the receiver operating characteristic (ROC) analysis. Besides the area under the ROC curve (AUC), accuracy, sensitivity, specificity, positive predictive value, negative predictive value, and F1-score were also calculated using the threshold determined by maximizing Youden-index in the training cohort. Furthermore, decision curve analysis^,[20,21]^ and calibration curve^[22]^ were used to assess the diagnostic performance of the model. The impact of variables on the treatment response of LRTIs was investigated using SHapley Additive exPlanations (SHAP).^[23,24]^ The SHAP analysis provides valuable interpretability to our predictive model by quantifying the contribution of each feature to the risk of treatment failure. By examining SHAP values, we can identify which patient characteristics and clinical parameters most significantly influence the model’s predictions. This transparency into the model’s decision-making process enables clinicians to understand the underlying factors driving the risk assessment. Such insights are instrumental in clinical practice, as they help identify high-risk patients who may benefit from tailored interventions. SHAP analysis not only enhances the interpretability of the model but also facilitates personalized treatment strategies, supporting clinicians in making informed decisions to improve patient outcomes. The overall workflow chart is illustrated in Figure 1.

## 3. Results and discussion

### 3.1. Characteristics of study cohort

A total of 790 patients with LRTIs were initially collected. After 44 patients who failed to meet the inclusion criteria were excluded, 746 patients were included for analysis (Figure S1, Supplemental Digital Content, https://links.lww.com/MD/P484). Among them, 631 patients were categorized into the treatment-effective group, while 115 patients were classified into the treatment-ineffective group. The baseline characteristics of all participants are detailed in the supplemental materials (Table S1, Supplemental Digital Content, https://links.lww.com/MD/P484).

### 3.2. Feature selection

**Figure 2.**
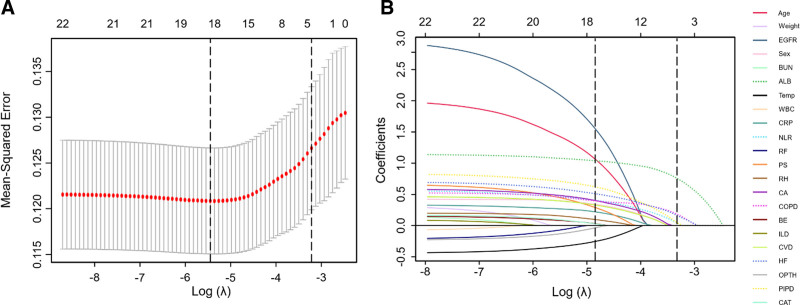
(A) Plot of the relationship between the cross-validation error and the *log*-transformed penalty parameter (*λ*) in the LASSO regression analysis. The left and right vertical dashed lines denote the *λ* value corresponding to the minimum cross-validation error and the maximum *λ* value whose corresponding cross-validation error is within 1 standard error from the minimum error, respectively. (B) LASSO regression screening path for the variables based on the optimal *log* of λ. LASSO = least absolute shrinkage and selection operator.

The variation of 5-fold cross-validation error with the log-transformed penalty parameter (*λ*) in LASSO regression is presented in Figure 2A. Following the 1-standard-error rule,^[25]^ the selected log(*λ*) value is approximately −3.3. With the selected *λ*, 5 features (ALB, NLR, COPD, HF, and dosage of PIPT) with nonzero coefficients were retained for further model building, as shown in Figure 2B.

### 3.3. Model evaluation and selection

**Figure 3.**
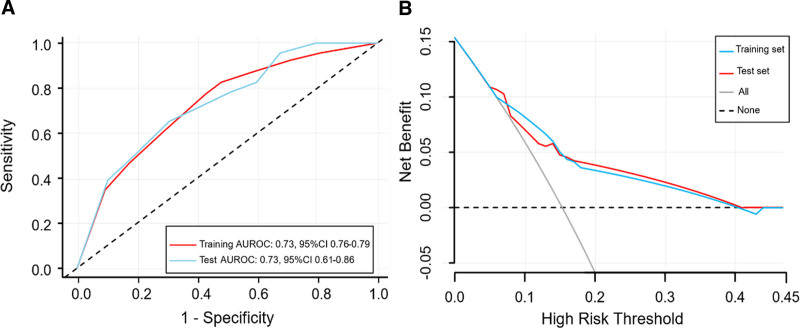
Performance of the DT model: (A) ROC curves, (B) decision curve on test set. DT = decision tree, ROC = receiver operating characteristic.

After feature selection, hyperparameter optimization was performed using grid search (version 1.3.0, https://scikit-learn.org/stable/modules/generated/sklearn.model_selection.GridSearchCV.html), facilitating the development of the ML models. Detailed hyperparameters of all models are shown in Table S2, Supplemental Digital Content, https://links.lww.com/MD/P484. In the test set, the AUC values for LR, DT, RF, Gradient Boosting Decision Tree and XGBoost models were 0.72 (95% CI: 0.59–0.84), 0.73 (95% CI: 0.61–0.86), 0.74 (95% CI: 0.62–0.86), 0.67 (95% CI: 0.54–0.80), and 0.71 (95% CI: 0.59–0.84), respectively. DeLong tests revealed no significant differences between the RF, LR, and DT models (*P* > .05) (Table S3, Supplemental Digital Content, https://links.lww.com/MD/P484). Given that all features and the target variable are binary, the DT model was chosen for its interpretability and computational efficiency, despite the marginally higher AUC of the RF model. (Table S4, Supplemental Digital Content, https://links.lww.com/MD/P484) provides a comprehensive overview of the performance metrics for all models built in this study. The ROC curve and decision curve for the DT model are shown in Figures 3A and B. The decision curve analysis demonstrates that the DT model offers a higher net benefit compared to the “Treat all” and “Treat none” strategies when the predicted probability is in the range of 0.05 to 0.40, highlighting its practical utility in clinical decision-making. Consequently, a predictive model based on the DT was selected for further analyses.

### 3.4. Model explanation

Risk estimates can be extracted from the DT model by evaluating SHAP values to allow interpretation of risk on the patient or global level. By calculating the average absolute SHAP values for each feature on the test set, a global importance ranking of features was obtained. The bar plot in the figure illustrates the relative importance of each feature in predicting treatment response (Figure S2, Supplemental Digital Content, https://links.lww.com/MD/P484).

**Figure 4.**
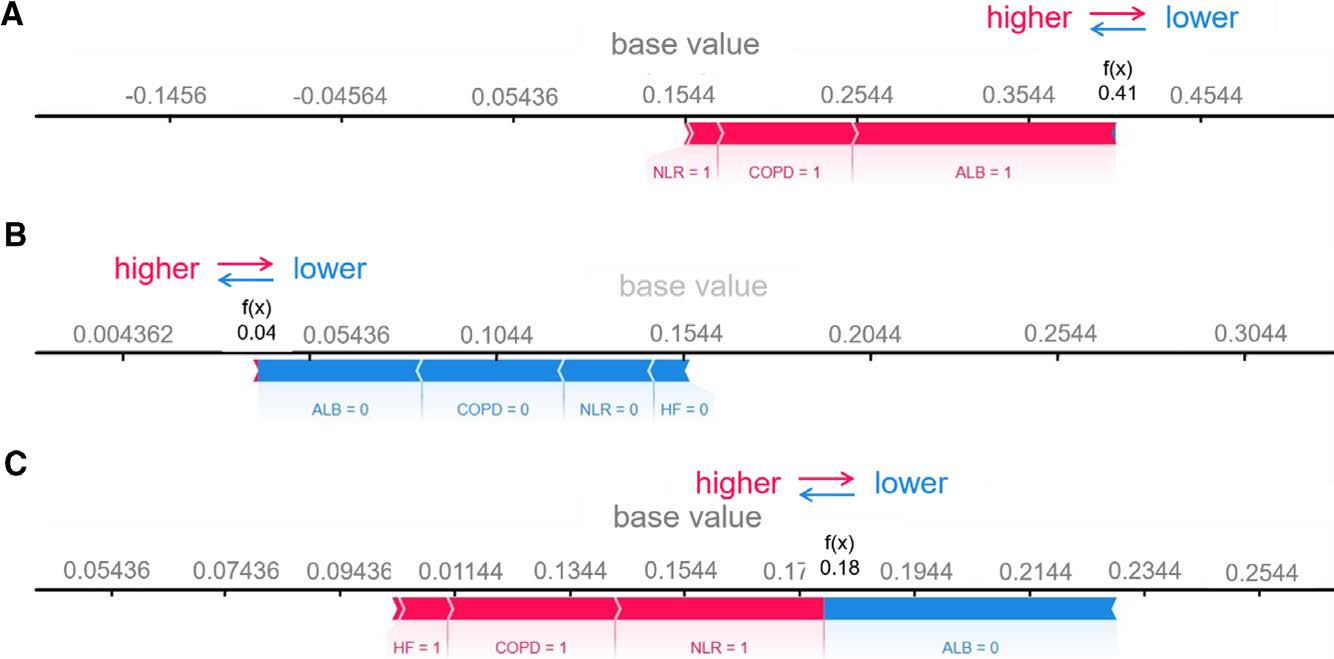
Contribution of features in patients with the (A) highest, (B) lowest and (C) moderate predicted SHAP values. SHAP = SHapley Additive exPlanations.

To further elucidate the DT model, we conducted an analysis of individual-level risk predictions and their respective risk factors identified by the SHAP values using the DT model. For the patient with the highest predicted SHAP value (i.e., 0.41), ALB = 1, COPD = 1, and NLR = 1 are identified as the contributing risk factors leading to the elevated SHAP value (Fig. 4A). Conversely, for the patient with the lowest SHAP value (i.e., 0.04, ALB = 0, COPD = 0, NLR = 0, and HF = 0) are observed (Fig. 4B). Additionally, for a patient with a moderate SHAP level (−1.76), the predictors contributing to a higher SHAP value (i.e., HF = 1, COPD = 1, NLR = 1) are counterbalanced by predictors leading to a lower SHAP value (ALB = 0) (Fig. 4C).

In this study, we combined ML with SHAP interpretability methods to develop and validate a ML model for predicting the treatment efficacy of PITT for LRTIs and investigated the key factors influencing the outcome. Five clinical features, including ALB, COPD, NLR, HF, and dosage of PIPT were identified as relevant to the effectiveness of PIPT.

SHAP force plots demonstrate a clear correlation between ALB levels and treatment failure risk with PIPT. Specifically, when ALB is below 35 g/L (ALB = 1), the risk of treatment failure increases. Conversely, when ALB is at or above 35 g/L (ALB = 0), the risk decreases. ALB serves as a crucial indicator for assessing patient nutrition and disease severity.^[26,27]^ As an important transport protein, ALB is responsible for delivering antibiotics to the site of infection, thereby enhancing their therapeutic effects.^[28]^ Lower ALB concentrations in the blood before treatment can affect the delivery and distribution of antibiotics at the infection site, reducing their concentration and impacting efficacy.^[29]^ When ALB levels are low, the transport and distribution of antibiotics are impeded, leading to a reduced concentration at the infection foci and subsequently lowering treatment effectiveness. Furthermore, insufficient albumin normally indicates malnutrition, which can disrupt antibiotic absorption and metabolism, thereby reducing treatment efficacy. Malnutrition can also weaken immune function, reducing the body’s ability to combat infection and diminishing the efficacy of antibiotic therapy.^[30]^ Nutrient levels, such as salts, metals, and vitamins in body fluids, are significantly different mechanisms and pathways in the risk of PIPT treatment failure from ALB.^[31–33]^

In addition to ALB levels, SHAP analysis highlights the significant impact of COPD and HF on PIPT efficacy. The force plots identify patients with HF (HF = 1) and COPD (COPD = 1) as critical risk factors that elevate SHAP values. Patients with COPD often exhibit airway inflammation and obstruction, which can reduce the delivery and concentration of antibiotics in the lungs.^[34,35]^ Patients with HF commonly experience reduced cardiac function and fluid retention,^[36]^ leading to pulmonary congestion and edema. Pulmonary congestion increases lung fluid volume, resulting in denser lung tissue,^[37]^ which in turn lowers the concentration of antibiotics at the site of infection, thereby diminishing treatment efficacy. Furthermore, pulmonary edema can impair lung function by reducing the activity and quantity of immune cells, compromising the body’s ability to resist infection and making it more challenging to eradicate infections.

Moreover, SHAP force plots indicate that a high NLR (NLR ≥ 75%, denoted as NLR = 1) is a significant risk factor, contributing to increased SHAP values. NLR is a crucial biomarker for assessing the inflammatory status and immune function of the body, directly influencing the efficacy of antibiotic therapy for LRTIs.^[38]^ A high NLR signifies an overactive inflammatory response to infection, which increases the activity of immune cells and may lead to greater inflammatory damage.^[39]^ In such cases, the effectiveness of antibiotics is reduced, as overactivated immune cells can disrupt the antimicrobial action of antibiotics or make bacteria more resistant to clearance.^[40]^ Additionally, an elevated NLR indicates a decline in immune function, potentially leading to less effective treatment outcomes.

Although this study achieved acceptable predictive results, it is important to acknowledge its limitations. Firstly, this is a study using data from one institution; further multi-center studies should be warranted to improve and validate the model. Secondly, our model was specifically developed for PIPT, focused on its efficacy in cases of bacterial LRTIs. However, this approach can also be extended to other types of LRTIs. Furthermore, it can be applied to cancer diagnosis, treatment, drug selection, and prognosis evaluation.^[41,42]^ Lastly, despite our efforts to minimize bias through rigorous inclusion and exclusion criteria, the retrospective nature of data collection can introduce inherent limitations, such as missing data and potential selection bias. Therefore, larger sample sizes and longitudinal prospective studies are necessary to further validate the effectiveness and applicability of our predictive model in real-world clinical settings.

## 4. Conclusion

In conclusion, we developed a DT model to predict the therapeutic efficacy of PIPT in treating bacterial LRTIs. Combined with SHAP interpretability analysis, our model can expose the impact of each feature on predicting outcomes, which enhances its interpretability and utility in clinical settings. Our model demonstrated its potential as a valuable tool for clinicians to optimize antibiotic treatment decisions and facilitate personalized treatment for LRTI patients.

## Author contributions

**Data curation:** Zhijing Zhu, Tao Yang, Yemeng Yang.

**Methodology:** Zhijing Zhu, Kun Han, Yemeng Yang, Likun Pan.

**Software:** Zhijing Zhu, Tao Yang, Kun Han, Xinjuan Liu.

**Visualization:** Tao Yang, Kun Han, Xinjuan Liu, Yemeng Yang, Likun Pan.

**Writing – original draft:** Zhijing Zhu, Tao Yang, Kun Han.

**Writing – review & editing:** Xinjuan Liu, Yemeng Yang, Likun Pan.

## Supplementary Material


