# Supplementary material for: Interpretable prediction on treatment response of piperacillin–tazobactam for lower respiratory tract infections using machine learning
Source: Medicine (Baltimore). 2025 Aug 1;104(31):e43460. doi: 10.1097/MD.0000000000043460 (PMC12323956; doi:10.1097/MD.0000000000043460)
Supplement: Supplementary file 1 [file medi-104-e43460-s001.docx]

**Supplementary Material**

**Table S1** Baseline characteristics of the patients.

| Characteristics | Total (N = 746) | Effective (n=631) | Ineffective (n=115) | *P-Value* |
| --- | --- | --- | --- | --- |
| Gender, No. (%) |  |  |  |  |
| Male = 1 | 497 (66.6) | 410 (65.0) | 87 (75.7) | 0.03 |
| Female = 0 | 249 (33.4) | 221 (35.0) | 28 (24.3) |  |
| Age, median (IQR), y | 73.0 (65.5-80.5) | 73.0 (65.0-80.0) | 78.0 (68.5-83.0) | <.001 |
| Weight, median (IQR), kg | 61.0 (52.5-69.5) | 61.0 (53.0-70.0) | 60.0 (50.5-70.0) | .30 |
| EGFR^[[1]](#footnote-1)^, median (IQR), % | 89.5 (75.7-98.9) | 89.1 (75.9-99.0) | 89.8 (75.1-97.5) | .24 |
| BUN^[[2]](#footnote-2)^, No. (%) |  |  |  |  |
| <11 mmol/L = 0 | 680 (91.2) | 576 (91.3) | 104 (90.4) | .90 |
| >=11 mmol/L = 1 | 66 (8.8) | 55 (8.7) | 11 (9.6) |  |
| ALB^[[3]](#footnote-3)^, No. (%) |  |  |  |  |
| >=35 g/L = 0 | 553 (74.1) | 496 (78.6) | 57 (49.6) | <.001 |
| <35 g/L = 1 | 193 (25.9) | 135 (21.4) | 58 (50.4) |  |
| Temp^[[4]](#footnote-4)^, No. (%) |  |  |  |  |
| <37.3 °C = 0 | 569 (76.3) | 478 (75.8) | 91 (79.1) | 0.51 |
| >=37.3 °C = 1 | 177 (23.7) | 153 (24.2) | 24 (20.9) |  |

| WBC^[[5]](#footnote-5)^, No. (%) |  |  |  |  |
| --- | --- | --- | --- | --- |
| <9.5*10^(-9) = 0 | 498 (66.8) | 428 (67.8) | 70 (60.9) | 0.18 |
| >=9.5*10^(-9) = 1 | 248 (33.2) | 203 (32.2) | 45 (39.1) |  |
| CRP^[[6]](#footnote-6)^, No. (%) |  |  |  |  |
| <10.0 mg/L = 0 | 349 (46.8) | 308 (48.8) | 41 (35.7) | 0.01 |
| >=10.0 mg/L = 1 | 397 (53.2) | 323 (51.2) | 74 (64.3) |  |
| NLR^[[7]](#footnote-7)^, No. (%) |  |  |  |  |
| <70 % = 0 | 344 (46.1) | 306 (48.5) | 38 (33.0) | .003 |
| >=70 % = 1 | 402 (53.9) | 325 (51.5) | 77 (67.0) |  |
| RF^[[8]](#footnote-8)^, No. (%) |  |  |  |  |
| NO = 0 | 546 (73.2) | 465 (73.7) | 81 (70.4) | .54 |
| YES = 1 | 200 (26.8) | 166 (26.3) | 34 (29.6) |  |
| PS^[[9]](#footnote-9)^, No. (%) |  |  |  |  |
| NO = 0 | 60 (8.0) | 55 (8.7) | 5 (4.3) | .16 |
| YES = 1 | 686 (92.0) | 576 (91.3) | 110 (95.7) |  |
| RH^[[10]](#footnote-10)^, No. (%) |  |  |  |  |
| NO = 0 | 636 (85.3) | 547 (86.7) | 89 (77.4) | .02 |
| YES = 1 | 110 (14.7) | 84 (13.3) | 26 (22.6) |  |

| CA^[[11]](#footnote-11)^, No. (%) |  |  |  |  |
| --- | --- | --- | --- | --- |
| NO = 0 | 623 (83.5) | 536 (84.9) | 87 (75.7) | .02 |
| YES = 1 | 123 (16.5) | 95 (15.1) | 28 (24.3) |  |
| COPD^[[12]](#footnote-12)^, No. (%) |  |  |  |  |
| NO = 0 | 339 (45.4) | 306 (48.5) | 33 (28.7) | <.001 |
| YES = 1 | 407 (54.6) | 325 (51.5) | 82 (71.3) |  |
| BE^[[13]](#footnote-13)^, No. (%) |  |  |  |  |
| NO = 0 | 592 (79.4) | 505 (80.0) | 87 (75.7) | .35 |
| YES = 1 | 154 (20.6) | 126 (20.0) | 28 (24.3) |  |
| ILD^[[14]](#footnote-14)^, No. (%) |  |  |  |  |
| NO = 0 | 669 (89.7) | 566 (89.7) | 103 (89.6) | .01 |
| YES = 1 | 176 (23.6) | 38 (21.9) | 38 (33.0) |  |
| CVD^[[15]](#footnote-15)^, No. (%) |  |  |  |  |
| NO = 0 | 570 (76.4) | 493 (78.1) | 77 (67.0) | .01 |
| YES = 1 | 176 (23.6) | 38 (21.9) | 38 (33.0) |  |
| HF^[[16]](#footnote-16)^, No. (%) |  |  |  |  |
| NO = 0 | 421 (56.4) | 374 (59.3) | 47 (40.9) | <.001 |
| YES = 1 | 325 (43.6) | 257 (40.7) | 68 (50.1) |  |

| OPTH^[[17]](#footnote-17)^, No. (%) |  |  |  |  |
| --- | --- | --- | --- | --- |
| NO = 0 | 106 (14.2) | 87 (13.8) | 19 (16.5) | .53 |
| YES = 1 | 640 (85.8) | 544 (86.2) | 96 (83.5) |  |
| PIPD^[[18]](#footnote-18)^ |  |  |  |  |
| 3.125g, Q8H = 0 | 360 (48.3) | 315 (49.9) | 45 (39.1) | .04 |
| 2.5g, Q8H = 1 | 386 (51.7) | 316 (50.1) | 70 (60.9) |  |
| CAT^[[19]](#footnote-19)^, No. (%) |  |  |  |  |
| NO = 0 | 389 (52.1) | 333 (52.8) | 56 (48.7) | .48 |
| YES = 1 | 357 (47.9) | 298 (47.2) | 59 (51.3) |  |

**Table S2** Hyperparameters used to build all models.

| Model | Hyperparameters |
| --- | --- |
| Logistic Regression | max_iter=2000,  random_state=0 |
| Decision Tree | max_depth=4,  min_samples_split=92,  min_samples_leaf=1,  max_features='sqrt',  random_state=0 |
| Random Forest | n_estimators=200,  max_depth=3,  min_samples_split=2,  min_samples_leaf=30,  max_features='sqrt',  random_state=0 |
| Gradient Boosting Decision Tree | n_estimators=200,  max_depth=3,  min_samples_leaf=1,  min_samples_split=2,  learning_rate=0.01,  random_state=0 |
| eXtreme Gradient Boosting | n_estimators=150,  max_depth=3,  learning_rate=0.16,  min_child_weight = 5,  subsample = 0.6,  colsample_bytree = 0.6,  gamma = 0.1,  reg_alpha = 1,  reg_lambda =1,  random_state=0 |

**Table S3** DeLong tests between the models.

| *P-Value* | Logistic Regression | Decision Tree | Gradient Boosting Decision Tree | | eXtreme Gradient Boosting |  |
| --- | --- | --- | --- | --- | --- | --- |
| Random Forest | 0.253 | 0.900 | 0.024 | 0.129 | | |

**Table S4** Performance metrics for the ML models on test set.

|  | AUC (95%CI) | Accuracy | SE | SP | PPV | NPV | F1-score | Cutoff |
| --- | --- | --- | --- | --- | --- | --- | --- | --- |
| LR | 0.72 (0.59-0.84) | 0.66 | 0.83 | 0.63 | 0.29 | 0.95 | 0.43 | 0.15 |
| DT | 0.73 (0.61-0.86) | 0.69 | 0.65 | 0.69 | 0.27 | 0.92 | 0.39 | 0.18 |
| RF | 0.74 (0.62-0.86) | 0.66 | 0.83 | 0.63 | 0.29 | 0.95 | 0.43 | 0.15 |
| GBDT | 0.67 (0.54-0.80) | 0.73 | 0.57 | 0.76 | 0.30 | 0.91 | 0.39 | 0.21 |
| XGBoost | 0.70 (0.57-0.82) | 0.63 | 0.73 | 0.61 | 0.25 | 0.93 | 0.38 | 0.13 |


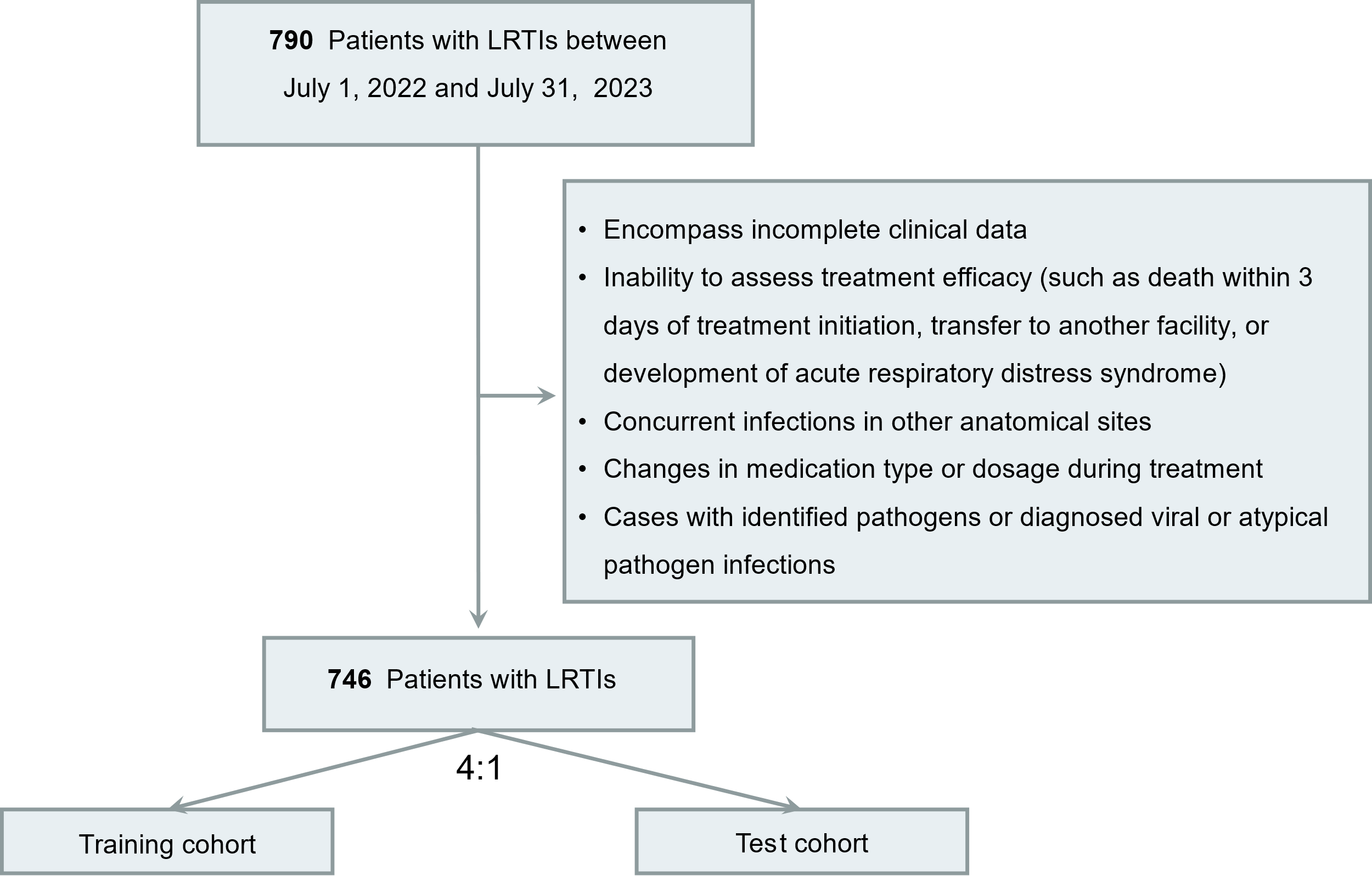
**Figure. S1.** Selection process of the study population.


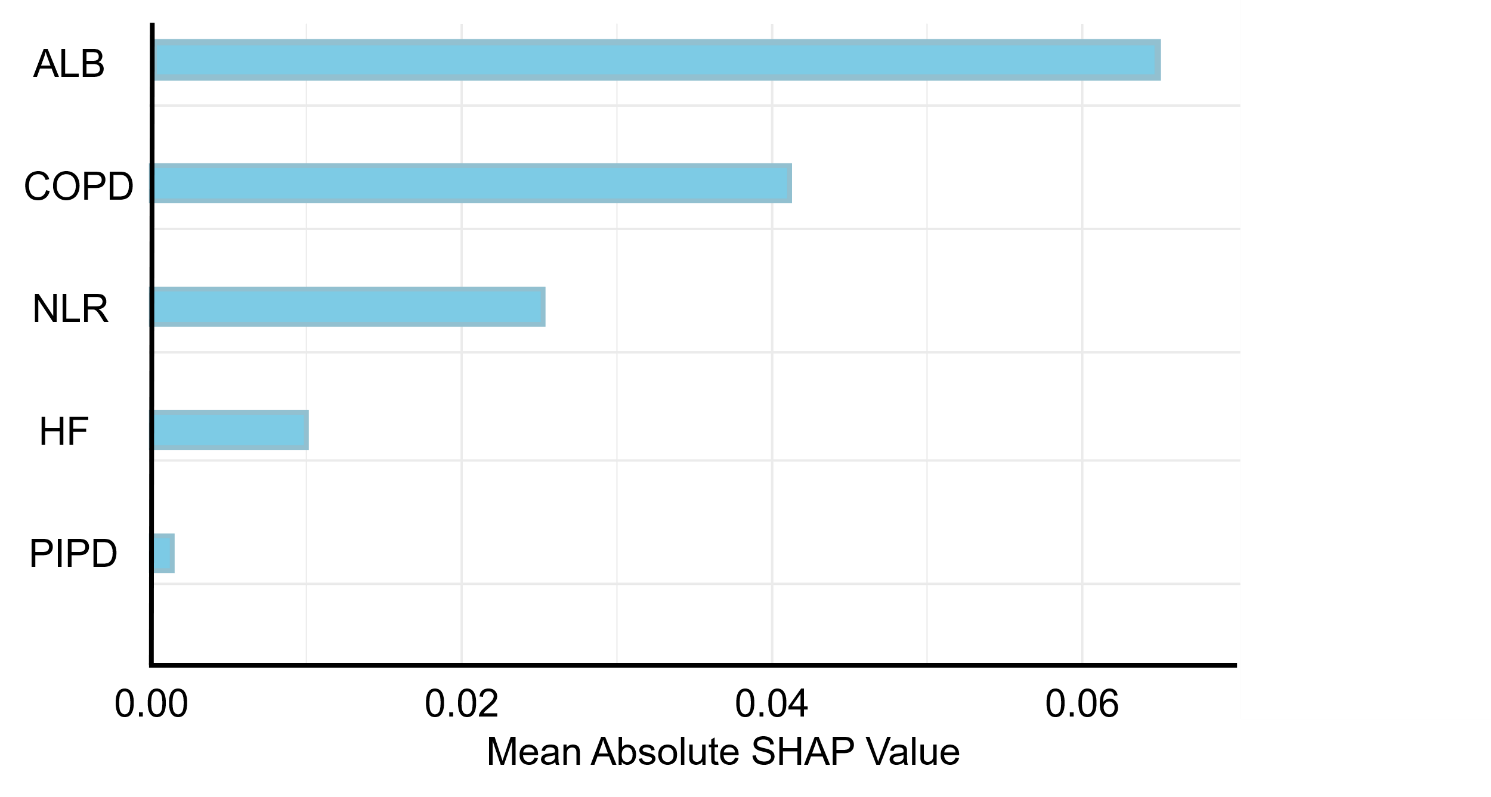


**Figure. S2.** Feature importance bar chart for the LR Model displaying average absolute SHAP values.

1. EGFR: Estimated glomerular filtration rate [↑](#footnote-ref-1)
2. BUN; Blood urea nitrogen [↑](#footnote-ref-2)
3. ALB: Serum Albumin [↑](#footnote-ref-3)
4. Temp: Body Temperature [↑](#footnote-ref-4)
5. WBC: White Blood Cell Count [↑](#footnote-ref-5)
6. CRP: C-Reactive Protein [↑](#footnote-ref-6)
7. NLR: Neutrophil-to-Lymphocyte Ratio [↑](#footnote-ref-7)
8. RF: Respiratory Failure [↑](#footnote-ref-8)
9. PS: Purulent sputum [↑](#footnote-ref-9)
10. RH: Recent hospitalization history(in 1 month) [↑](#footnote-ref-10)
11. CA: Active tumor [↑](#footnote-ref-11)
12. COPD: Chronic Obstructive Pulmonary Disease [↑](#footnote-ref-12)
13. BE: Bronchiectasis [↑](#footnote-ref-13)
14. ILD: Interstitial lung disease [↑](#footnote-ref-14)
15. CVD: Cerebrovascular disease [↑](#footnote-ref-15)
16. HF: Heart Failure [↑](#footnote-ref-16)
17. OPTH: Outpatient treatment history before admission [↑](#footnote-ref-17)
18. PIPD: Dosage of Piperacillin/Tazobactam [↑](#footnote-ref-18)
19. CAT: Combination antimicrobial therapy [↑](#footnote-ref-19)
